# Design and Implementation of Digital Calibration Certificate for RFID Tag Storage

**DOI:** 10.3390/s24206626

**Published:** 2024-10-14

**Authors:** Kan Kan, Shuaizhe Wang, Zilong Liu, Xingchuang Xiong

**Affiliations:** 1 Guangdong Provincial Institute of Metrology (South China National Centre of Metrology), Guangzhou 510405, China; kankan@scm.com.cn; 2 National Institute of Metrology, Beijing 100029, China; wangshuaizhe@nim.ac.cn (S.W.); liuzl@nim.ac.cn (Z.L.); 3 Key Laboratory of Metrology Digitalization and Digital Metrology for State Market Regulation, Beijing 100029, China

**Keywords:** metrology, calibration, digitalization of metrology, digital calibration certificate, RFID

## Abstract

The digital transformation of metrology is one of the most active activities in the field of metrology at present, where the digital calibration certificate (DCC) being developed is the topic receiving the most concern in the current digital transformation. In practical industrial applications, the issue of storage carrier for the DCC plays a crucial role in its promotion and implementation. To address this issue, a DCC meta-model called DCC-Lite schema has been designed along with a corresponding processing method. This solution involves compressing and segmenting the DCC to make it suitable for storage using RFID tags. These RFID tags are affixed to the instruments and accompany them throughout their usage. Additionally, the DCC-RFID processing system has been developed to validate the effectiveness of the DCC meta-model and its corresponding processing method within a wireless temperature acquisition system. Experimental results demonstrate that the system successfully reads and writes the DCC stored within the RFID tag group. Furthermore, it enables automated parsing of the DCC calibration data by the machine and real-time compensation of measurement data to reduce measurement errors.

## 1. Introduction

In recent years, digital transformation of metrology has received continuous attention from metrology and industry, leading to significant growth in related research fields [1,2]. The digital transformation of metrology is an important part of the CIPM (International Committee for Weights and Measures) Strategy 2030+ [3]. In 2022, during the 27th CGPM (General Conference on Weights and Measures), the adoption of the resolution “on Global Digital Transformation and the International System of Units”—CGPM Resolution 2 (2022)—encouraged the CIPM to establish and promote the SI digital framework as an anchor of trust in metrology in the digital world [4]; subsequently, a “Joint Statement of Intent—On the Digital transformation in the international scientific and quality infrastructure” [5] was signed with various international organizations such as ISO, IEC, ISC, etc., highlighting the importance of digital transformation efforts in the metrology community.

The results of a CIPM survey on digital transformation [6] reveal that among the different digital transformation topics, digital calibration certificates (DCCs) were the most mentioned topic. Calibration is one of the important activities in the field of metrology, and it is a technical means to realize the traceability of quantity and value [7]. Calibration is particularly important in numerous areas, in particular in industry, where it is an important pillar for correct and comparable measurements and manufacturing in industry [8]. However, the currently prevalent use of paper-based and electronic calibration certificates suffers from limitations in data interoperability and reusability during the process of industrial digitalization. In contrast, DCCs possess machine-readable capabilities and contain data that can be easily and efficiently parsed, allowing for automated processing by machines. This enables companies to fully leverage the data contained within the calibration certificate, thereby creating significant added value for those utilizing DCCs [9].

The storage carrier of DCCs is a critical factor that impacts the widespread adoption of DCC applications. There are roughly two types of solutions for storing DCCs: storing them in a network server or storing them directly in the instrument or its control system. DCCs are designed to be machine-readable, making them particularly suitable for transmission and storage within a network. Consequently, DCC applications based on network transmission have been extensively studied in relevant literature [10,11,12,13,14,15]. However, relying solely on network storage may not be applicable in all DCC application scenarios. For instance, the network of measurement devices is often isolated for security and other considerations, which poses a hindrance to the circulation of DCCs. Furthermore, many measuring devices lack the storage function necessary for storing DCCs. If DCCs are stored in the control computer of the calibrated instrument, this requires adaptation and modification to accommodate the diverse communication interfaces and control systems found in different types of calibrated instruments. These challenges pose obstacles to the versatility and ease of use of the system. Given that DCCs serve as proof of traceability for measuring instruments and quantitatively demonstrate their measuring capabilities, finding a suitable storage carrier to establish a physical-level connection between DCCs and measuring instruments can enhance the correspondence between them. This, in turn, will enrich the utilization of DCCs and promote their wider application.

RFID technology has found extensive applications in various industries, including agriculture, logistics, and manufacturing [16,17,18,19], and its advantages of read-write and non-contact are very suitable for use as the storage carrier of DCCs for measuring instruments, However, there are certain limitations, such as the limited tag capacity, that hinder its direct use as the storage carrier for existing DCCs. In order to develop a user-friendly DCC storage carrier, we have undertaken the following work, taking into consideration the specific characteristics of DCC application in the industry and the current state of RFID technology:

1. Propose DCC meta-model DCC-Lite suitable for RFID tag storage, and, at the same time, take the means of compression and storing DCCs in tag groups to solve the problem of limited RFID tag storage space;
2. Develop DCC-RFID processing system to realize the process in 1;
3. Develop a wireless temperature acquisition system, together with the DCC-RFID app, to realize the whole process of applying DCCs with RIFD tags as a storage carrier in wireless temperature acquisition scenarios and verify the effectiveness of the system by comparing it with the measurement data of a reference temperature sensor with higher accuracy.

The rest of this paper is organized as follows: Section 2 will introduce the overall design of the RFID tag as a storage carrier for DCCs; Section 3 introduces the principle and method of implementation; Section 4 introduces the implementation process of DCCs with RFID tags as a storage carrier in the application scenario of a wireless temperature acquisition system and designs a comparative experiment to validate the effectiveness of the system; Section 5 analyzes the results of the experiment and discusses the results of the calibration of the DCC according to its data compensation of real-time measurement data; Section 6 provides conclusions.

## 2. Design

### 2.1. Design Principles and Application Scenarios

The purpose of utilizing RFID tags as a storage carrier for DCCs is to facilitate machine-to-machine (M2M) communication and interoperability. This enables the streaming of calibration data to production sites and allows machines at industrial sites to process real-time measurements or perform other automated operations based on the calibration data. Manual involvement in the processing of data within the calibration certificate is eliminated. When designing a DCC suitable for RFID tag storage, the following principles should be considered:

- The DCC designed for storage in RFID tags should be compatible with the general DCC format and capable of successful parsing by standard DCC parsing programs;
- The storage solution should be applicable to most measurement equipment. It should require minimal modification of existing equipment, enabling the seamless integration of DCCs at the physical level;
- The storage program should try to use common technology and tools, minimizing the technical barriers involved in implementation.

**Figure 1:**
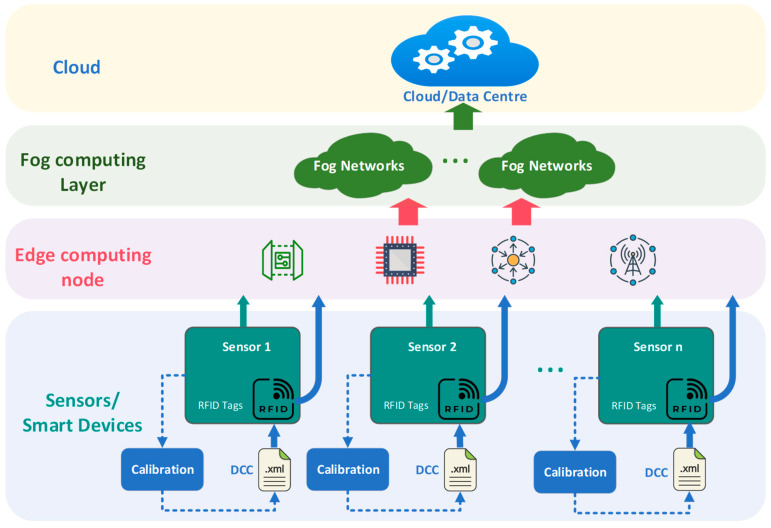
Application scenarios—sensor networks.

The digital transformation of metrology is primarily driven by the emerging requirements resulting from the digitalization of industries. In Industry 4.0, DCC will serve as a unified data medium connecting data applications (e.g., digital twins), industrial control systems, and field sensors, which will be able to rule out transmission errors, and the calibration results are immediately available for further processing. The realization of Industry 4.0 requires comprehensive process awareness, which poses new challenges for measurement and data acquisition. Massive sensors are interconnected through the IIoT (Industrial Internet of Things) [20]. In order to cope with the pressure on the cloud brought about by the large number of sensors’ data acquisition, the measurement data from the field will be computed at appropriate nodes closer to the field through the edge layer and the fog network layer and aggregated in the cloud. The DCC is stored in an RFID tag affixed to the sensors, and the data acquisition system can read the DCC file in the RFID tags, so as to effectively utilize the calibration data. The RFID tag as a DCC carrier in the sensor network application scenario is shown in Figure 1.

### 2.2. Design of DCC-Lite Meta-Model

Currently, there are two prominent formats for high-impact DCCs: the PDF/A3-based DCC, proposed by the Swiss national metrology institute (METAS) [21,22,23], and the XML-based DCC, coordinated by the Physikalisch-Technische Bundesanstalt (PTB) [24,25].

**Figure 2:**
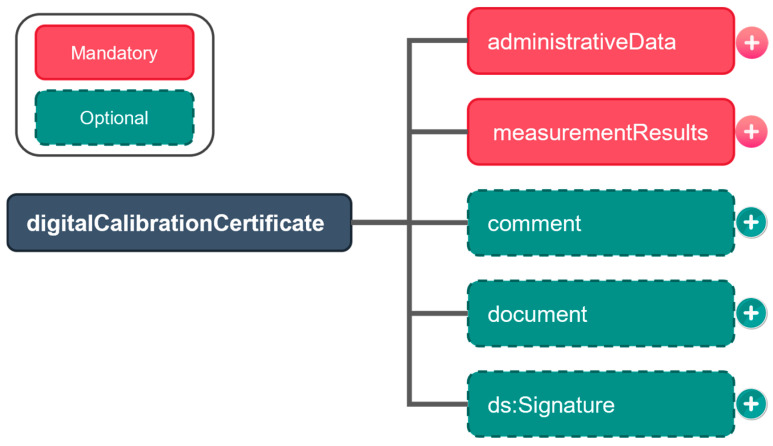
Root element structure of PTB-coordinated digital calibration certificate (DCC) schema (3.2.1).

The PTB-coordinated DCC, provided in the Extensible Markup Language (XML), is a highly popular solution for DCCs [1,26], and the DCC discussed in this paper is a PTB-coordinated DCC. The DCC depended on a standalone schema (dcc.xsd), which consists of a root element and a tree-like structure. The current version of the DCC’s XML schema (3.2.1) has a root element called “digitalCalibrationCertificate”, which contains five child elements: administrativeData, measurementResults, comment, document, and signature; its structure is shown in Figure 2.

By analyzing the structure of the DCC schema, it can be seen that the structure of the DCC schema contains optional human-readable content [27] in addition to content specifically designed for machine-readability. In a fully machine-interactive application scenario, these human-readable contents have limited value but consume significant storage space resources.

As demonstrated by the published example in GP (Good Practice), the DCC with a PDF document added to the “dcc:document” element for enhanced human readability can occupy as much as 2 MB of storage space [28]. Conversely, a DCC with less human-readable content can occupy as little as 16 kb [29]. However, the storage capacity of RFID tags commonly used in industrial settings is limited. Storing a complete DCC within a single RFID tag poses challenges, particularly when dealing with substantial amounts of human-readable content. Currently, high-frequency RFID tags in the industry typically have a single-chip capacity of up to about 8000 bytes [30]. Although higher capacity tags can be customized, they come at a significantly higher cost.

**Figure 3:**
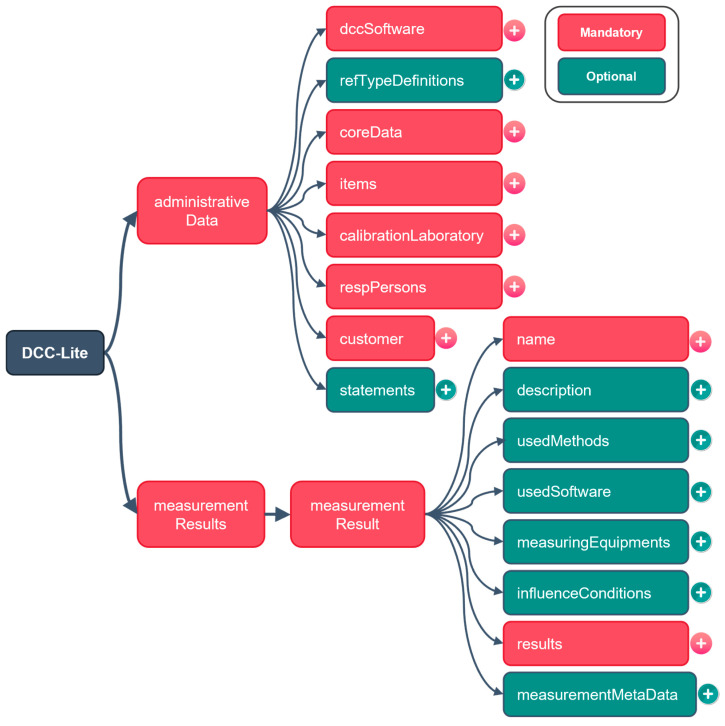
Structure of DCC-Lite.

Considering both economy and efficiency, the relevant contents primarily intended for human readability are cropped from the DCC schema structure. The key elements, such as “dcc:administrativeData” and “dcc:measurementResults”, are retained to generate a DCC meta-model suitable for storage on RFID tags (referred to as DCC-Lite schema). This DCC-Lite is used in conjunction with the complete DCC. The cropped portions mainly involve the human-readable section, which is a non-mandatory element of the DCC metadata model and does not affect the legitimacy validation of the DCC. The primary data elements of the DCC-Lite meta-model are illustrated in Figure 3.

### 2.3. Key Technologies

#### 2.3.1. Compression

Compared to other information exchange formats (e.g., JSON), XML has a drawback of data redundancy due to the presence of numerous duplicate tags, resulting in a significant increase in volume. For instance, each XML element requires a start tag (in the format “<element name>”) and an end tag (in the format “</element name>”). These tags must exist in pairs, meaning that for every start tag, there must be a corresponding end tag. Sometimes the length of formatting characters exceeds the length of the transmitted data.

For DCCs (XML format files) that contain a lot of redundant content, compression algorithms are suitable to reduce the file size to fit the application scenarios with constraint resources. Considering the generality of the technology, the open source compression library Zlib [31] can be utilized, and the DCC processing library released by Siemens—PyDCC (PyDCC was licensed under the open-source license MIT), which contains the compression function module, and the principle of implementation is to refer to Zlib to achieve the compression of the DCC [32].

**Figure 4:**
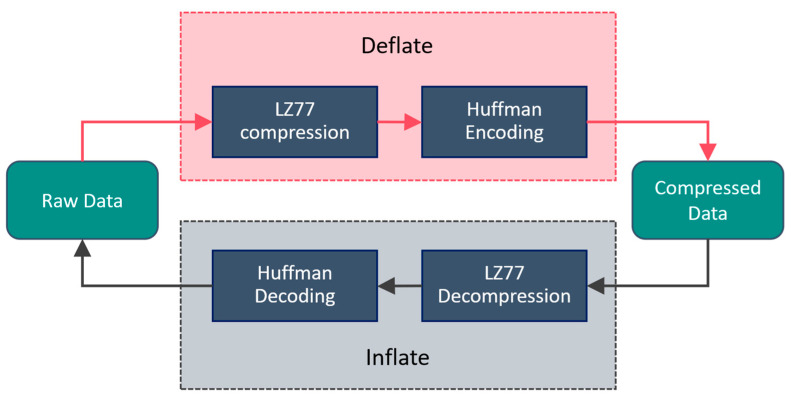
Zlib compression and decompression algorithm implementation process.

The compression algorithm employed by Zlib is the Deflate algorithm [33], which is a lossless compression algorithm based on the LZ77 compression algorithm with Huffman coding. The decompression process is the reverse of the compression process. The principles of Zlib’s compression and decompression algorithms are illustrated in Figure 4.

The LZ77 algorithm, proposed by Abraham Lempel and Jacob Ziv in 1977, is a data compression technique that utilizes dictionaries. Its main concept involves exploiting the repetitive structural information within the data for compression purposes. Specifically, it aims to identify whether the sequence of characters being compressed has appeared in previously processed data. If so, the algorithm represents the recurring characters using the encoding of offsets and lengths of the character sequence. The compression effectiveness improves with higher data repetitiveness. In our case, we employ the Zlib library, as demonstrated in the published GP (Good Practice) example [34]; for compression, the results are shown in Table 1. It is evident that, on average, Zlib achieves a compression rate of approximately 80% for DCC files. However, when it comes to DCCs containing PDF files (where the PDF content is converted to Base64 encoding and placed within the “document” section of the DCC), the compression rate is lower, around 25%.

#### 2.3.2. Use RFID Tag Group

Another approach to address the issue of limited RFID capacity is by utilizing groups of RFID tags. When generating DCCs based on the DCC-Lite meta-model, it is possible that the storage space of a single RFID tag may not be sufficient to accommodate larger volumes of DCC data. In such cases, multiple RFID tags can be grouped together to store the DCC.

**Figure 5:**
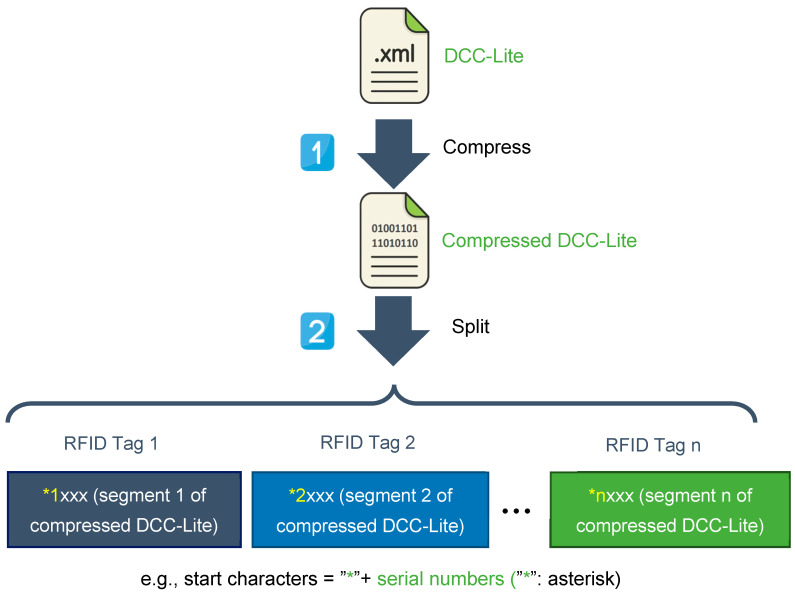
Process of DCC-Lite writing to RFID tags group.

DCCs are divided into several segments based on the capacity of the tags. At the beginning of each segment, a start character is added, which is then written into the corresponding tag. This inclusion of the start character aids in the subsequent reading and splicing operations. The schematic diagram illustrating the storage of DCC-Lite using groups of RFID tags is depicted in Figure 5.

## 3. Implementation

### 3.1. Generation of DCC-Lite

The National Metrology Data Center (NMDC) at the National Institute of Metrology, China (NIM), has established a microservice-based DCC system, which includes an online generation wizard. This system includes an online generation wizard [35] with wizard-based DCC generation and DCC verification; it provides a RESTful data interface API [36] that allows users to generate DCCs by making calls through the interface. The interaction with the interface occurs using the JSON data format.

Though the above basic tools can generate DCC files, the generated DCC file is in accordance with the DCC-Lite structure described in Figure 1. During the generation process, three non-mandatory sub-elements (“comment”, “document”, and “ds:Signature”) are removed from the root element to create the DCC-Lite. This transformation is made possible due to the machine-readable nature of the XML format, enabling easy and automated execution by machines.

### 3.2. Write DCC-Lite to RFID Tags

The method of writing DCC-Lite into an RFID tag group is as follows: Firstly, the DCC-Lite file is compressed using the LZ77 compression and Huffman encoding algorithms (realized by invoking the Zlib library), obtaining the storage space capacity occupied by the compressed DCC-Lite. Subsequently, considering the storage capacity of the RFID tags and the capacity occupied by the start character, the required number of RFID tags can be calculated based on Formula (1).

(1) $$n=\left\lceil \frac{v_{d}}{\left(v_{r}-v_{c}\right)}\right\rceil$$

- n: Number of RFID tags required, n is an integer, round up to an integer;
- $v_{d}$: Capacity occupied by DCC-Lite after compression, in bytes;
- $v_{r}$: Capacity of a single RFID tag, in bytes;
- $v_{c}$: Capacity used by the start character, in bytes.

The process of writing DCC-Lite into an RFID tag group involves several steps. Firstly, the compressed DCC-Lite is divided into n segments (data blocks) based on the capacity of a single RFID tag. Each segment is prefixed with a start sign to ensure that, except for the last segment, each segment’s capacity, including the start sign, matches that of a single RFID tag.

Careful consideration should be given to the design of the start character, which should include a sequence number for each segment (e.g., the start character can be designed as asterisk + sequence number, such as *1, *2). This design allows for easy concatenation of content read from each RFID tag during subsequent operations.

Before writing data to the RFID tags, the existing data on the tags need to be cleared. Then, the segmented data are written to each RFID tag using an RFID reader/writer.

The specific steps for writing DCC-Lite to an RFID tag group are as follows:

1. Compress the DCC-Lite (XML) using Zlib;
2. Calculate the capacity of the compressed DCC-Lite, the capacity of a single RFID tag, and the capacity of start characters;
3. Calculate the required number of RFID tags, rounded up to an integer;
4. Split the compressed DCC-Lite into n data blocks and add start characters at the beginning of each data block and the capacity of each data block (start characters included), except the last one is the RFID tag capacity;
5. Clear the contents of the RFID tags;
6. Write data blocks to the corresponding RFID tags sequentially through an RFID reader/writer.

### 3.3. Read DCC-Lite from RFID Tags

The method of reading data segments from RFID tags and assembling them into a complete DCC-Lite is as follows: Firstly, all the RFID tags on the measuring instrument are read through the RFID reader and saved in sequential order. Then, based on the sequence numbers contained in the start characters at the beginning of each data segment, the data segments are arranged in ascending order, and the start characters of each segment are removed. Subsequently, the data segments, now without the start characters and arranged in ascending order, are spliced together. The Huffman Decoding and LZ77 Decompression algorithms (implemented by invoking the Zlib library) are then used to decompress the data. Finally, the decompressed data are assembled into the complete DCC-Lite file.

The specific steps for reading out DCC-Lite from an RFID tag group are as follows:

1. Read each RFID tag on the measurement instrument using an RFID reader/writer and save them individually;
2. Extract the serial numbers of the tags from the start characters and delete the start characters. Arrange the data blocks in ascending order;
3. Assemble each data block to form a complete compressed DCC-Lite file;
4. Decompress the DCC-Lite file using the Zlib library.

## 4. Testing and Validation

### 4.1. Test System Setup

The test system is a wireless temperature acquisition system for real-time monitoring of laboratory equipment temperature and ambient temperature. Each temperature logger is equipped with a Wi-Fi transmission module, allowing it to automatically connect to a preconfigured wireless router and establish a TCP/IP connection with a server port on the local area network (LAN). Data are transmitted in JSON format, and the loggers can also receive parameter setting commands in JSON format. The use of Wi-Fi enables wireless transmission of measurement data, providing flexibility in instrument layout and avoiding the need for rewiring when equipment is relocated.

**Figure 6:**
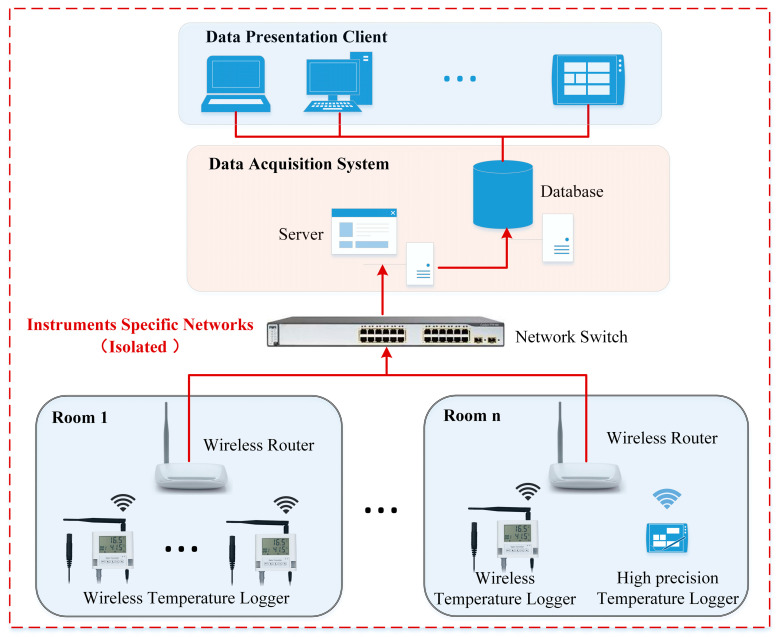
Architecture of test system.

To ensure security management, the system utilizes an isolated LAN specifically designed for instrument use. This LAN is physically separated from the office network and the internet. A data acquisition system based on .Net (C#) is employed to receive real-time JSON packets transmitted by the wireless temperature sensors. The system parses and stores the temperature data in a database and provides services such as temperature overrun alarms. A client program is developed for data display, allowing computers or tablets within the isolated LAN to view real-time and historical temperature data. The architecture of the test system is depicted in Figure 6.

The system utilizes a wireless temperature logger with a maximum permissible error (MPE) of ±0.5 °C. These loggers are equipped with digital temperature sensors (DS18B20). In addition, the system incorporates high-precision temperature loggers that utilize platinum thermal resistors (PT100) as their temperature sensors. The high-precision temperature loggers offer a maximum permissible error (MPE) of ±0.1 °C.

**Figure 7:**
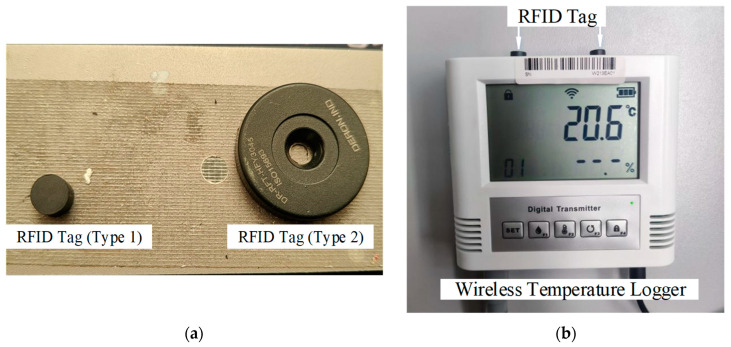
(**a**) RFID tag used in the system; (**b**) RFID tags affixed to the wireless temperature logger.

The RFID tag group is attached to the temperature logger, allowing for the storage of the DCC of the temperature logger within the tag. This enables the transfer of the DCC along with the instrument and facilitates non-contact reading through an RFID reader. The system offers various options for the types and forms of RFID tags to accommodate different instruments. The RFID tags utilized in this system are depicted in Figure 7a. Type 1 tags have a diameter of only 8 mm, while Type 2 tags have a thickness of approximately 3 mm. Both types of tags are high-frequency (13.56 MHz) RFID tags and have a capacity of 2k bytes. They are used in conjunction with corresponding high-frequency RFID readers/writers, having a reading and writing distance of approximately 5 cm. The RFID readers/writers communicate with the computer via the Modbus/RTU protocol for data exchange.

### 4.2. Sensor DCC Write into RFID Tags

**Figure 8:**
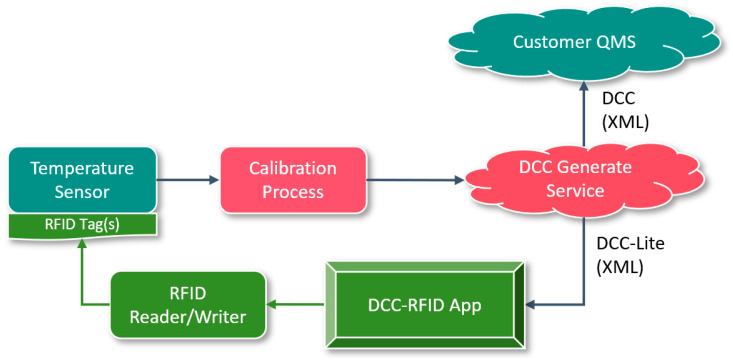
The realization process of DCC generation and storing RFID tags.

The wireless temperature logger utilized in the system undergoes calibration, and the DCC generate service generates DCC and DCC-Lite according to the calibration data. The generated DCC is either pushed to or made accessible through an interface to the customer quality management system via the DCC platform for retrieval. On the other hand, the generated DCC-Lite is compressed and written into the RFID tag group attached to the measuring device using an RFID reader/writer. This facilitates the seamless flow of DCC alongside the measuring device and instrument. The implementation process is illustrated in Figure 8.

### 4.3. Sensor DCC Read and Run

Once the temperature logger has been calibrated and returned to the site, the DCC-RFID App is employed to control the RFID reader/writer to read the contents stored within the RFID tag group affixed to the temperature logger. The app performs various operations such as splicing, decompressing, and restoring the contents back into the DCC-Lite file. Furthermore, it parses the calibration data present within the DCC-Lite file. Table 2 displays the calibration data for one of the wireless temperature sensors (device number: W215JD) within the system.

Based on the calibration data and compensation model, the temperature acquisition system generates compensation data, which is then stored in the system’s database. During the operation of the temperature acquisition system, it retrieves the compensation data for the corresponding wireless temperature logger from the database. Upon receiving measurement data from the wireless temperature logger, the system calculates the corresponding compensation value based on the real-time measurement value and the data compensation curve. This compensation value is then applied to the real-time data, enabling real-time compensation of the measurement data. The client data display program can be utilized to observe the real-time measurement data before and after compensation.

To account for the characteristics of the wireless temperature logger that employs a digital temperature sensor, data compensation is conducted using piecewise linear fitting based on the compensation values obtained from calibration points. The compensation model is represented by Formula (2).

(2) $$\begin{array}{l} x_{c}=x_{m}+y_{c}\\ y_{c}=\left(\frac{y_{n}-y_{n-1}}{x_{n}-x_{n-1}}\right)\left(x_{m}-x_{n-1}\right)+y_{n-1},x_{m}\in \left[x_{n-1}\left.,x_{n}\right)\right. \end{array}$$

**Figure 9:**
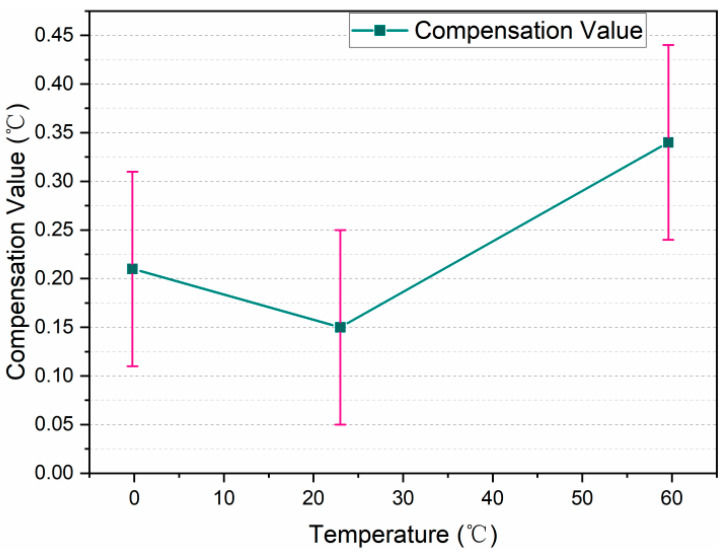
Wireless temperature sensor (W215JD) data compensation curve.

- x_c_: The measured value after compensation of the temperature logger;
- x_m_: The measured value obtained from the temperature logger;
- y_c_: The compensation value corresponding to x_m_;
- x_n_: The temperature value of the calibration point n, where n is an integer not less than 2;
- y_n_: The compensation value corresponding to the calibration point n;

The compensation curve calculated according to the calibration data of the wireless temperature logger whose device number is W215JD in Table 2 is shown in Figure 9.

It is important to highlight that various types of sensors possess distinct compensation models, which must be tailored to the characteristics of the instrument and relevant professional theories. It should be noted that not all sensors are suitable for direct compensation of measurement data using the calibration data in DCC without further processing.

**Figure 10:**
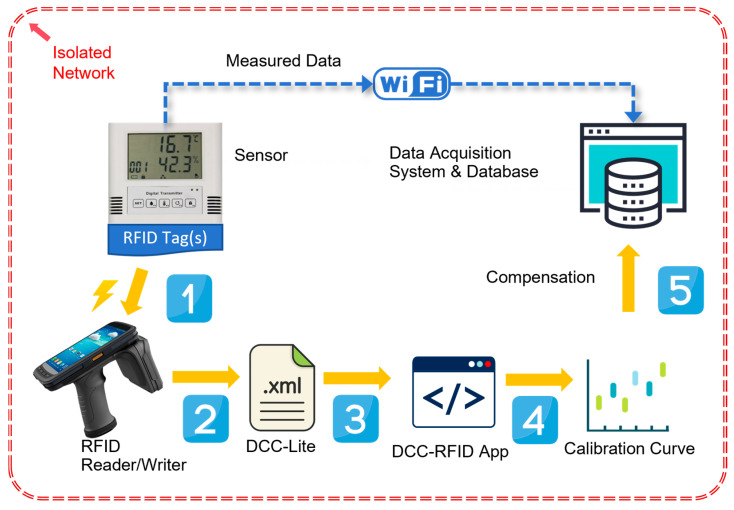
The system reads the DCC inside the RFID tag and compensates for the measurement data operation process.

Figure 10 illustrates the process of DCC and measurement data compensation through RFID tags processing within the system.

### 4.4. System Operation

To validate the effectiveness of measurement data compensation, a wireless temperature logger (MPE: ±0.5 °C) with device number W215JD was selected for a verification experiment within the system. The logger underwent calibration, and its corresponding DCC was stored in an RFID tag group affixed to its body. Upon returning to the field of use, with the help of DCC-RFID app, the flow of the DCC to the isolated local area network where the temperature acquisition system operates is facilitated.

**Figure 11:**
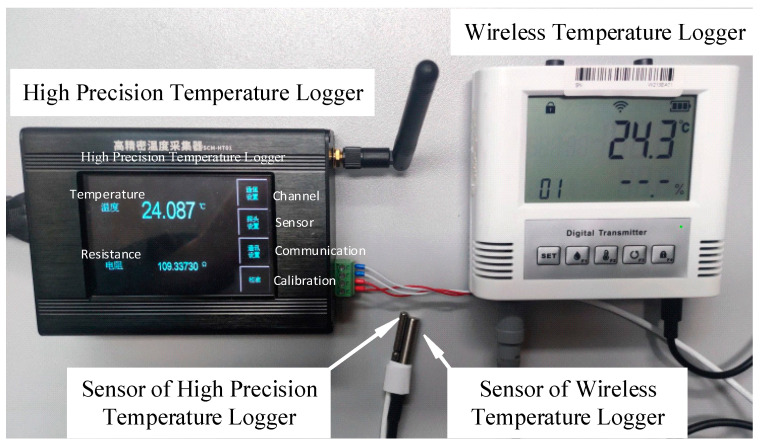
Sensors of high-precision temperature logger and wireless temperature logger are securely attached.

For comparison purposes, a high-precision temperature logger with an MPE of ±0.1 °C (equipment number: G00014) was utilized. The sensor probes of both loggers were securely attached and placed in the same temperature environment. The wireless temperature logger was configured to upload data every 2 min, and the system automatically collected the temperature data. The system performed real-time compensation of the measurement data obtained from the wireless temperature logger using the compensation parameters derived from the parsed calibration data within the DCC. The measurement data before and after compensation were recorded accordingly. Figure 11 illustrates the processing of the wireless temperature logger and the high-precision temperature logger sensors.

## 5. Results and Discussion

**Figure 12:**
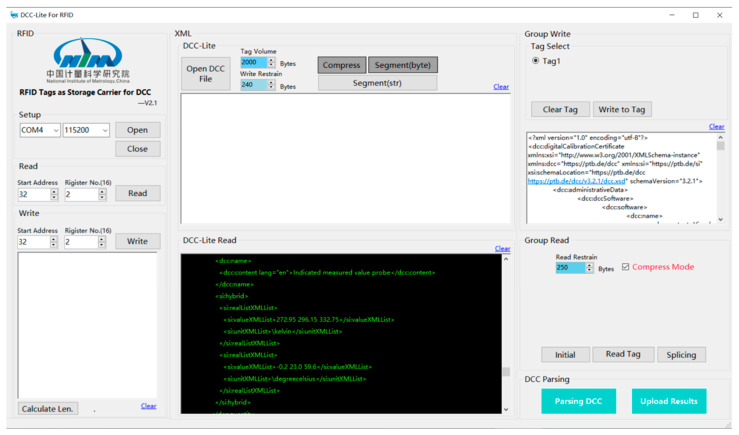
The UI of DCC-RFID app.

The body of the wireless temperature logger is equipped with two RFID tags, each with a capacity of 2000 bytes. The digital calibration certificate (DCC) is stored within this group of tags. The DCC-RFID app is utilized to read and parse the DCC from the RFID tag attached to the wireless temperature logger. The resulting compensation data are then stored in the database of the data acquisition system. Figure 12 illustrates the user interface of the DCC-RFID app during operation.

The Data Acquisition System is employed to collect the measurement data from the temperature logger within the isolated LAN continuously. The data collection occurs at regular intervals of 2 min.

**Figure 13:**
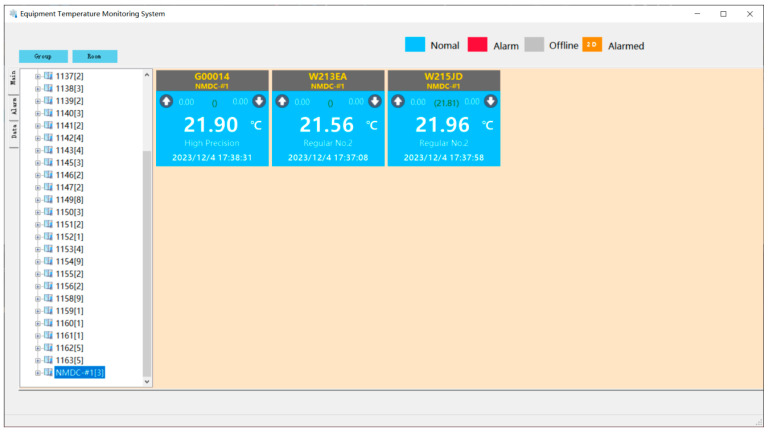
The UI of Data Presentation Client.

The collected measurement data can be accessed and viewed through the Data Presentation Client. Figure 13 showcases the user interface of the client program. The display frame presents the measurement data after compensation, while the data enclosed in square brackets represent the original data collected by the temperature logger before compensation.

Data from two specific time periods, namely 18:00–24:00 on 1 December 2023, and 09:00–15:00 on 4 December 2023, were selected for analysis. Both the wireless temperature logger (MPE: ±0.5 °C) and the high-precision temperature logger (MPE: ±0.5 °C) were included in the analysis.

**Figure 14:**
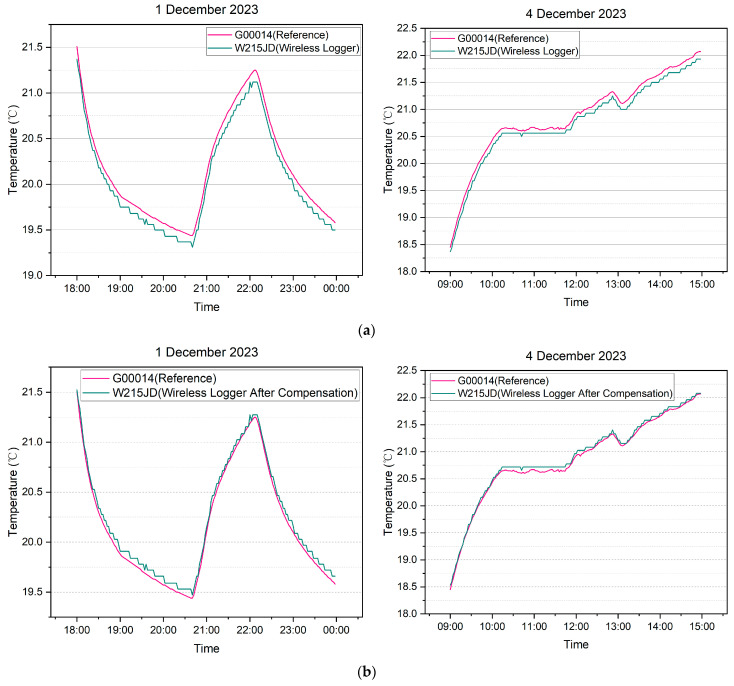
(**a**) Comparison between measurement data of wireless temperature logger (W215JD) and reference device before compensation; (**b**) Comparison between measurement data of wireless temperature logger (W215JD) and reference device after compensation.

Figure 14a presents a comparison of temperature data with an accuracy of ±0.1 °C. In Figure 14b, the temperature data from the wireless temperature logger is compared to that of the high-precision temperature logger after compensation by the system using the DCC calibration data within the same time period. Notably, it can be observed that the measurement data from the wireless temperature logger, after compensation, closely align with the data of the high-precision temperature logger.

**Figure 15:**
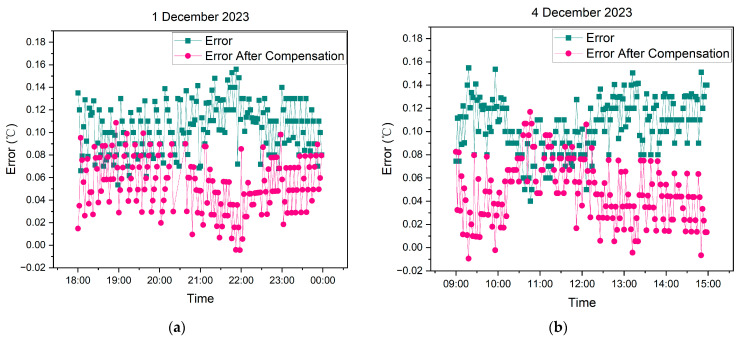
(**a**) Comparison of display errors of measurement data of wireless temperature logger (W215JD) before and after compensation (data date: 1 December 2023); (**b**) Comparison of display errors of measurement data of wireless temperature logger (W215JD) before and after compensation (data date: 4 December 2023).

Figure 15 illustrates the distribution of error for the measurement data from the wireless temperature logger before and after compensation. In this analysis, the high-precision temperature logger is considered as the standard reference.

Upon observing the error distribution, it becomes evident that the error is relatively concentrated within a specific range. Moreover, the overall error of the measurement data after compensation is lower compared to that before compensation.

The comparison of errors between the measurement data before and after compensation with the reference data is shown in Table 3.

As depicted in Table 3, it is evident that the system achieves a significant reduction in error when compensating the measurement data based on the calibration data within the DCC. For the 1 December 2023, dataset, the mean absolute error (MAE) decreases from 0.105 before compensation to 0.056 after compensation. Additionally, the root mean square error (RMSE) decreases from 0.107 before compensation to 0.060 after compensation. Similarly, for the 4 December 2023, dataset, the MAE decreases from 0.105 before compensation to 0.048 after compensation, while the RMSE decreases from 0.107 before compensation to 0.055 after compensation.

These experimental results demonstrate the feasibility of storing the DCC within the limited capacity RFID tag through using DCC-Lite schema, compression, and tag grouping methods. The DCC can be successfully read in a network isolated environment and can be automatically analyzed by the machine for real-time compensation of temperature acquisition data. The utilization of the calibration data within the DCC leads to a significant reduction in measurement errors.

## 6. Conclusions

DCCs, like traditional calibration certificates, need to follow a series of norms (such as ISO/IEC 17025 [37], SI brochure, VIM, GUM, etc.) to meet the requirements of recognition, mutual recognition, and metrological traceability. Unlike a mere digital representation of a paper calibration certificate, the DCC is designed to enable machine-to-machine interaction without human intervention. To achieve this, all formal and technical terms used in the DCC need to be semantically described, precisely defining and specifying the content of each element within the document. Therefore, the design of the DCC is an iterative process that requires careful discussion. The true value of the DCC lies in its ability to benefit stakeholders, particularly end users, more effectively than traditional calibration certificates. With its machine-readable and machine-interpretable characteristics based on the metrological semantic system, the DCC has broader applications in the digital world. Exploring practical application scenarios for the DCC holds significant reference value for its design.

We delve into the practical application of the DCC. To facilitate the circulation use of the DCC, we propose a method that employs RFID tags as storage carriers for the DCC. Addressing the meta-model of the DCC, we introduce a tailored version called DCC-Lite. This modified model resolves the issue of the limited storage capacity of RFID tags through XML file compression technology and an RFID tag group-based read-write mechanism. Additionally, we develop a related system and apply it to a wireless temperature acquisition system. The operational results of the system demonstrate that the DCC stored in the RFID tag can be easily transferred along with the measuring instrument and can be read offline, thereby addressing the application challenges of the DCC in data acquisition systems with network isolation requirements. This research provides a reference solution for the application of the DCC to measuring instruments lacking data interfaces or storage capabilities. The end-user system can automatically extract the calibration data from the DCC using machine reading techniques and utilize this data to compensate for real-time measurement data based on the characteristics of the measuring instrument, thereby reducing the instrument tolerance budget.

Due to the possibility of machine automated reading and processing of data in digital calibration certificates for industrial parameter adjustment, ensuring the integrity, authenticity, and tamper resistance of digital calibration certificates becomes more important. This paper explores the functional implementation of RFID tags as carriers for digital calibration certificates (DCC) and verifies their functionality but does not address security protection mechanisms. The security assurance mechanism for storing digital calibration certificates on RFID tags is an area that requires further research in future studies.

## 7. Patents

The research content of this article has been granted a patent in China. The patent is titled “A method for using RFID tags as carriers for digital calibration certificates and its system” with the patent number “ZL 2023 1 1505359.X” [38].

## Figures and Tables

**Table 1:** Comparison of Zlib’s Compression Effect on GP Example DCCs.

GP Name	Volume before Compress (Bytes)	Volume after Compress (Bytes)	Compress Ratio (%)
dcc_gp_temperature_simplified_v12	15,538	3529	77.3
dcc_gp_temperature_typical_v12	19,688	4464	77.3
dcc_gp_temperature_typical_adjustment_v12	24,675	4697	81.0
dcc_gp_temperatur_resistance_v12	20,920	4889	76.6
dcc_gp_temperature_extensive_v12	32,154	5550	82.7
dcc_gp_humidity_v1.0	34,531	6614	80.8
dcc_gp_humidity_v1.0_with_hr_html	100,994	21,428	78.8
dcc_gp_humidity_v1.0_with_hr_pdf	2,054,591	1,534,460	25.3

**Table 2:** Calibration data of wireless temperature sensor (W215JD).

Indication Value (°C)	Reference Value (°C)	Compensation Value (°C)	MPE (°C)	Expanded Uncertainty (°C)
−0.2	0.01	0.21	±0.5	0.1
23.0	23.15	0.15	±0.5	0.1
59.6	59.94	0.34	±0.5	0.1

**Table 3:** Comparison of error before and after compensation of measurement data of wireless temperature sensor.

Data		MAE	MSE	RMSE
1 December 2023	Measured Value	0.105	0.011	0.107
	After Compensation	0.056	0.004	0.060
4 December 2023	Measured Value	0.105	0.012	0.107
	After Compensation	0.048	0.003	0.055

## Data Availability

The data presented in this study are available on request from the corresponding author.

## References

[1] Barbosa C.R.H., Sousa M.C., Almeida M.F.L., Calili R.F. (2022). Smart Manufacturing and Digitalization of Metrology: A Systematic Literature Review and a Research Agenda. Sensors.

[2] Hanisch R., Chalk S., Coulon R., Cox S., Emmerson S., Sandoval F., Forbes A., Frey J., Hall B., Hartshorn R. (2022). Stop squandering data: Make units of measurement machine-readable. Nature.

[3] BIPM Report on the Actions Taken by the CIPM towards a “CIPM Strategy 2030+”. https://www.bipm.org/documents/20126/17315032/CIPM-Strategy-2030.pdf/0870523b-cd7e-568e-a96a-adf78ff9edde?t=1664547939013.

[4] BIPM Resolution 2 of the 27th CGPM (2022). https://www.bipm.org/en/cgpm-2022/resolution-2.

[5] BIPM Joint Statement of Intent on the Digital Transformation in the International Scientific and Quality Infrastructure. https://www.bipm.org/documents/20126/42177518/Joint-Statement-Digital-Transformation.pdf.

[6] Werhahn O., Dudle G. Evaluation Report—Survey on Digital Transformation. https://www.bipm.org/documents/20126/82507584/RapportBIPM-2023-01.pdf/ff5aac7a-56d7-4ff7-7de1-04d024fedb65.

[7] BIPM International Vocabulary of Metrology—Basic and General Concepts and Associated Terms (VIM) 3rd Edition. https://www.bipm.org/documents/20126/2071204/JCGM_200_2012.pdf/f0e1ad45-d337-bbeb-53a6-15fe649d0ff1.

[8] Hackel S., Härtig F., Schrader T., Scheibner A., Loewe J., Doering L., Gloger B., Jagieniak J., Hutzschenreuter D., Söylev-Öktem G. (2021). The fundamental architecture of the DCC. Meas. Sens..

[9] Engel T. (2023). GEMIMEG-II—How metrology can go digital. Meas. Sci. Technol..

[10] Mustapää T., Nummiluikki J., Viitala R. (2022). Digitalization of Calibration Data Management in Pharmaceutical Industry Using a Multitenant Platform. Appl. Sci..

[11] Lin M.-X., Hsieh T.-H. (2023). Geometric Error Parameterization of a CMM via Calibrated Hole Plate Archived Utilizing DCC Formatting. Appl. Sci..

[12] Mustapää T., Tunkkari H., Taponen J., Immonen L., Heeren W., Baer O., Brown C., Viitala R. (2022). Secure Exchange of Digital Metrological Data in a Smart Overhead Crane. Sensors.

[13] Mustapää T., Nikander P., Hutzschenreuter D., Viitala R. (2020). Metrological Challenges in Collaborative Sensing: Applicability of Digital Calibration Certificates. Sensors.

[14] Bruns T., Nordholz J., Röske D., Schrader T. (2021). A demonstrator for measurement workflows using digital calibration certificates (DCCs). Meas. Sens..

[15] Nummiluikki J., Saxholm S., Kärkkäinen A., Koskinen S. (2023). Digital Calibration Certificate in an industrial application. Acta IMEKO.

[16] Černilová B., Linda M., Kuře J., Hromasová M., Chotěborský R., Krunt O. (2024). Validation of an IoT System Using UHF RFID Technology for Goose Growth Monitoring. Agriculture.

[17] Liu G., Wang Q.-A., Jiao G., Dang P., Nie G., Liu Z., Sun J. (2023). Review of Wireless RFID Strain Sensing Technology in Structural Health Monitoring. Sensors.

[18] Naciri L., Gallab M., Soulhi A., Merzouk S., Di Nardo M. (2023). Digital Technologies’ Risks and Opportunities: Case Study of an RFID System. Appl. Syst. Innov..

[19] Mulloni V., Marchi G., Gaiardo A., Valt M., Donelli M., Lorenzelli L. (2024). Applications of Chipless RFID Humidity Sensors to Smart Packaging Solutions. Sensors.

[20] Hackel S., Schönhals S., Doering L., Engel T., Baumfalk R. (2023). The Digital Calibration Certificate (DCC) for an End-to-End Digital Quality Infrastructure for Industry 4.0. Science.

[21] Boschung G., Wollensack M., Zeier M., Blaser C., Hof C., Stathis M., Blattner P., Stuker F., Basic N., Grasso Toro F. (2021). PDF/A-3 solution for digital calibration certificates. Meas. Sens..

[22] Phuaknoi P., Mongkolsuttirat K., Chantawong N., Limthunyalak W., Buajarern J. Implementation of Digital CalibrationCertificate at NlMT. Proceedings of the 2nd DCC-Conference 2022.

[23] Yamazawa K. Development of PDF based Digital Calibration Certificates at NMlJ, AIST. Proceedings of the 3rd DCC-Conference 2023.

[24] Gadelrab M.S., Abouhogail R.A. (2021). Towards a new generation of digital calibration certificate: Analysis and survey. Measurement.

[25] Hacker S.G., Härtig F., Hornig J., Wiedenhöfer T. The Digital Calibration Certificate. https://oar.ptb.de/files/download/310.20170403.pdf.

[26] Hackel S., Schönhals S., Gloger B., Doering L., Jagieniak J., Demir M.-A., Jordan M., Söylev Öktem G., Loewe J., Mienert K. Digitization Versus Digitalization & Digital Transformation in the Field of Calibrationsand Their Subsequent Use. https://oar.ptb.de/resources/show/10.7795/120.20240126.

[27] Demir M.-A., Jordan M., Krah T., Schönhals S., Hackel S., Härtig F., Schrader T., Loewe J., Doering L., Gloger B. A HUMAN READABLE FORM OF THE DCC. Proceedings of the First International IMEKO TC6 Conference on Metrology and Digital Transformation.

[28] PTB dcc_gp_humidity_v1.0_with_hr_pdf.xml. https://dccwiki.ptb.de/goodpractice/humidity/dcc_gp_humidity_v1.0_with_hr_pdf.xml.

[29] PTB dcc_gp_temperature_simplified_v12.xml. https://dccwiki.ptb.de/goodpractice/temperature/dcc_gp_temperature_simplified_v12.xml.

[30] ifm. ID Tags. https://www.ifm.cn/cn/en/category/220_030_020_030_070#/best/1/100.

[31] Roelofs G. A Massively Spiffy Yet Delicately Unobtrusive Compression Library. https://www.zlib.net/.

[32] Simens. pydcc. https://github.com/siemens/pydcc.

[33] Feldspar A. An Explanation of the Deflate Algorithm. https://www.zlib.net/feldspar.html.

[34] PTB Good Practice-Digital Calibration Certificate Wiki. https://dccwiki.ptb.de/en/gp_home.

[35] NIM NIM DCC Generation System. https://dcc.nmdc.ac.cn/dcc-web/wizard/.

[36] NIM DCC Base API. https://dcc.nmdc.ac.cn/dcc-doc/v1/dcc-base/swagger-ui/index.html.

[37] (2017). General Requirements for the Competence of Testing and Calibration Laboratories.

[38] Kan K., Xiong X., Fang X. (2024). A Method for Using RFID Tags as Carriers for Digital Calibration Certificates and Its System. ZL 2023 1 1505359.X.

